# Equations for smartphone prediction of adiposity and appendicular lean mass in youth soccer players

**DOI:** 10.1038/s41598-023-48055-y

**Published:** 2023-11-25

**Authors:** Marco A. Minetto, Angelo Pietrobelli, Andrea Ferraris, Chiara Busso, Massimo Magistrali, Chiara Vignati, Breck Sieglinger, David Bruner, John A. Shepherd, Steven B. Heymsfield

**Affiliations:** 1 Division of Physical Medicine and Rehabilitation, Department of Surgical Sciences, University of Turin, Turin, Italy; 2 Pennington Biomedical Research Centre, Baton Rouge, LA USA; 3 Department of Surgical Sciences, Dentistry, Gynaecology and Paediatrics, Paediatric Unit, University of Verona, Verona, Italy; 4 J|medical, Turin, Italy; 5 Juventus Football Club, Turin, Italy; 6 Size Stream LLC, Cary, NC USA; 7 Department of Epidemiology, University of Hawaii Cancer Center, Honolulu, HI USA

**Keywords:** Biomarkers, Nutrition, Whole body imaging

## Abstract

Digital anthropometry by three-dimensional optical imaging systems and smartphones has recently been shown to provide non-invasive, precise, and accurate anthropometric and body composition measurements. To our knowledge, no previous study performed smartphone-based digital anthropometric assessments in young athletes. The aim of this study was to investigate the reproducibly and validity of smartphone-based estimation of anthropometric and body composition parameters in youth soccer players. A convenience sample of 124 male players and 69 female players (median ages of 16.2 and 15.5 years, respectively) was recruited. Measurements of body weight and height, one whole-body Dual-Energy X-ray Absorptiometry (DXA) scan, and acquisition of optical images (performed in duplicate by the Mobile Fit app to obtain two avatars for each player) were performed. The reproducibility analysis showed percent standard error of measurement values < 10% for all anthropometric and body composition measurements, thus indicating high agreement between the measurements obtained for the two avatars. Mobile Fit app overestimated the body fat percentage with respect to DXA (average overestimation of + 3.7% in males and + 4.6% in females), while it underestimated the total lean mass (− 2.6 kg in males and − 2.5 kg in females) and the appendicular lean mass (− 10.5 kg in males and − 5.5 kg in females). Using data of the soccer players, we reparameterized the equations previously proposed to estimate the body fat percentage and the appendicular lean mass and we obtained new equations that can be used in youth athletes for body composition assessment through conventional anthropometrics-based prediction models.

## Introduction

Anthropometric measures are routinely assessed in sports and rehabilitative medicine because of their usefulness for performance optimization and (re-)injury prevention^1^. In fact, measurements such as the limb circumferences and skinfold thicknesses are widely used in young and adult athletes to assess the body composition (e.g., fat mass percentage, lean mass, muscle mass)^2–6^ and its training-related changes^7–11^. Because tape-based measurements of body circumferences and caliper-based measurements of skinfold thicknesses may not be culturally or socially acceptable and also exhibit poor reliability, especially in persons with overweight and obesity, there was the need for the development and validation of reproducible, valid, and cost-effective technologies suitable to perform non-invasive anthropometric and body composition assessments^12,13^. Digital anthropometry by three-dimensional optical imaging systems has recently been shown to provide non-invasive, precise, and accurate measurements^12–16^. However, three-dimensional imaging systems are still unavailable in most clinical and sports settings, whereas digital consumer cameras and smartphones (with their mobile applications using high-resolution imaging) became pervasive and offer new tools that can be used by physicians to evaluate patients and by exercise scientists to evaluate athletes. Tian et al.^17^ were the first to propose and validate an algorithm for predicting three-dimensional body shape and composition from a single frontal bi-dimensional image acquired with a digital consumer camera. Very recent studies performed in healthy adults showed that smartphone-based assessment of body circumferences^18–20^ and different body composition variables (i.e., body fat percentage, visceral adipose tissue, fat free mass, appendicular lean mass)^18,21–25^ was feasible (through two or four photographs) and produced reliable and valid (with respect to dual-energy X-ray absorptiometry—DXA^18,21,22,25^ or rapid four-compartment model^23,24^) measurements. These measurements can be particularly useful in young athletes to optimize the nutritional programs and to help identify underlying medical problems (e.g., eating disorders)^26^. However, no previous study, to our knowledge, performed smartphone-based digital anthropometric assessments in young athletes. Therefore, the aim of this study was to investigate the reproducibly and validity of smartphone-based estimation of clinically relevant anthropometric and body composition parameters in a large group of youth soccer players.

## Methods

### Participants and protocol

The study setting was a sports medicine and rehabilitation center where a convenience sample of 124 male soccer players [median age (1st–3rd quartile): 16.2 (15.0–18.4) years; body mass index: 21.4 (20.1–22.8) kg/m^2^] and 69 female soccer players [age: 15.5 (14.4–16.9) years; body mass index: 20.4 (19.6–21.4) kg/m^2^] were recruited to participate. Almost all (N = 181) players were Caucasian, with the exception of some (N = 12) African players. This single study visit was performed as part the preseason investigations and included measurements of body weight and height, whole-body DXA scan, and acquisition of optical images.

All subjects (or their parents in case of underage subjects) gave their written consent for study participation and publication of identifying information/images after receiving a detailed explanation of the protocol.

### Measurements

Body weight and height were measured while the subject was dressed in undergarments and with bare feet. Body weight and height were measured (to the nearest 0.1 kg and 0.5 cm, respectively) using a standard scale with stadiometer (model Seca 799, Seca GmbH & Co. Kg, Hamburg, Germany).

One whole-body DXA scan was performed on a Lunar iDXA system (GE Healthcare, Chicago, IL, USA) according to a standardized protocol. Duplicate DXA scans were not acquired to be radiation dose conserving. The output from the DXA scan included the following body composition measurements: (i) total fat mass and its percentage, (ii) total lean mass (i.e., whole-body soft lean mass plus bone mineral content), (iii) arms lean mass (i.e., the soft lean mass of the upper limbs), (iv) legs lean mass (i.e., the soft lean mass of the lower limbs), (v) appendicular lean mass (i.e., the sum of the soft lean mass of the upper and lower limbs).

**Figure 1:**
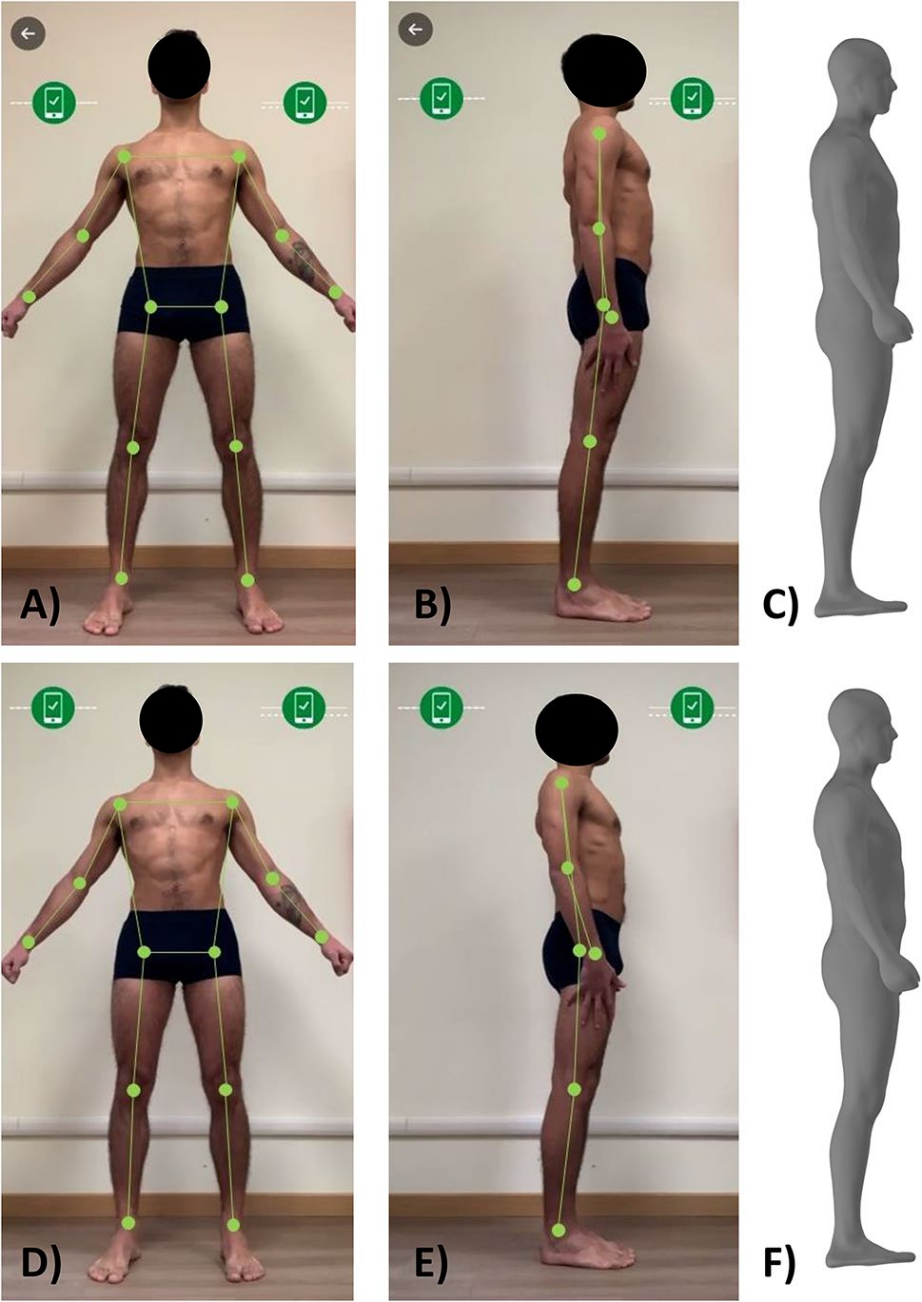
(**A**–**C**) representative example of acquisition of the frontal (**A**) and lateral (**B**) images in a male soccer player and the relative avatar (**C**) with the following measurements: average of the right and left arm circumference 32.3 cm, waist circumference: 87.9 cm, hip circumference: 100.8 cm, average of the right and left thigh circumference: 54.8 cm. (**D**–**F**) the improper placement of the upper limbs during the acquisition of the lateral image (**E**) in the same player of the (**A**,**B**) changed the shape of the avatar and biased the estimation of different body circumferences, as follows: average of the right and left arm circumference 30.7 cm, waist circumference: 81.5 cm (i.e., the waist circumference difference between the two avatars was ~ 6 cm), hip circumference: 99.3 cm, average of the right and left thigh circumference: 53.9 cm.

Optical images were taken with Mobile Fit app (version 3.0, Size Stream LLC, Cary, NC, USA) using a standardized positioning protocol. Voice commands from the app guided each player into position for the self-scan: as shown in Fig. 1A–D, the player was asked to assume a “front A-pose” (and to maintain the pose without movements of the trunk or limbs) to capture the frontal image. Next, the player was asked to assume a “side pose” (Fig. 1B–E) to capture the lateral image. After the image capture, the app software generated a de-identified 3D humanoid avatar (Fig. 1C–F) with associated anthropometric measurements and body composition estimates. The acquisition of the frontal and lateral images was performed in duplicate to obtain two avatars for each player. The image acquisition was further repeated if the experimenters noticed either movements of the trunk or limbs during the frontal image capture or the improper placement of the upper limbs and hands (as shown in a representative example in Fig. 1E) during the lateral image acquisition. In fact, body movements or an improper placement of the upper limbs can produce changes in the shape of the avatar (for example, the arm-blocking-back artefact and the hand-on-thigh artefact) that ultimately result in biased estimation of different body circumferences.

The Mobile Fit app report includes 243 whole-body and segmental circumferences, lengths, surface areas, and volumes. Body composition estimates can be derived from some of these measures using the previously published equations for estimation of the body fat percentage^27^ and appendicular lean mass^25^ (Table 1).

**Table 1:** Prediction equations for the body fat percentage and appendicular lean mass.

Variable	Equation	Refs
Body fat (% )	48.837 − 7.2745 × (1 for male, 0 for female) + 1.192 × (right thigh circ.) − 17.387 x ($\frac{\mathrm{right \; bicep\; circ}. +\mathrm{ left \;bicep \;circ}. +\mathrm{ right \; calf\; circ}. +\mathrm{ left \;calf \;circ}. +\mathrm{ right\; thigh \;circ}. +\mathrm{ left \; thigh \;circ}. }{\mathrm{maximum \; stomach \;circ}.}$)	^27^
	32.366 − 10.404 × (1 for male, 0 for female) + 0.461 × (right thigh circ.) − 11.755 × ($\frac{\mathrm{right \;bicep \; circ}. +\mathrm{ left \; bicep \;circ}. +\mathrm{ right \; calf \; circ}. +\mathrm{ left \; calf \; circ}. +\mathrm{ right \; thigh \; circ}. +\mathrm{ left \; thigh \; circ}. }{\mathrm{maximum \; stomach \; circ}.}$)	Current study (Eq. #1)
Appendicular lean mass (kg)—male subject	− 27.386 + 2.566 × (1 for NHOPI race, 0 for other races) + 0.132 × height + 0.221 × weight + 0.068 × (head circ.) + 0.087 × (chest circ.) + 0.212 × (forearm circ.) − 0.038 × (waist circ.) + 0.020 × (thigh circ.) − 0.024 × (outside leg length) + 0.0008 × (surface area arm) + 0.00005 × (arm volume) − 0.0002 × (torso volume) + 0.0003 × (leg volume)	^25^
	− 27.694 + 0.162 × height + 0.343 × weight − 0.531 × (head circ.) − 0.019 × (chest circ.) + 1.761 × (forearm circ.) − 0.008 × (waist circ.) + 0.178 × (thigh circ.) − 0.036 × (outside leg length) + 0.00038 × (surface area arm) − 0.00179 × (arm volume) − 0.0001 × (torso volume) + 0.0002 × (leg volume)	Current study (Eq. #2)
	− 25.012 + 0.123 × height + 0.348 × weight − 0.536 × (head circ.) + 1.998 × (forearm circ.) − 0.00185 × (arm volume) − 0.0001 × (torso volume)	Current study (Eq. #3)
Appendicular lean mass (kg)—female subject	− 12.622 − 0.023 × age − 0.191 × (1 for Caucasian race, 0 for other races) + 0.427 × (1 for Black race, 0 for other races) − 0.288 × (1 for Hispanic race, 0 for other races) + 0.553 × (1 for NHOPI race, 0 for other races) − 0.709 × (1 for Other race, 0 for Caucasian, Black, Asian, Hispanic, NHOPI, American Indian races) + 0.088 × height + 0.135 × weight + 0.001 × (head circ.) + 0.019 × (collar circ.) + 0.048 × (forearm circ.) + 0.078 × (upper arm circ.) − 0.039 × (waist circ.) + 0.083 × (ankle circ.) + 0.023 × (outside leg length) + 0.0003 × (surface area leg)	^25^
	0.467 × age − 22.183 × (1 for Caucasian race, 0 for other races) − 20.948 × (1 for Black race, 0 for other races) − 0.043 × height + 0.144 × weight + 0.121 × (head circ.) − 0.002 × (collar circ.) + 0.325 × (forearm circ.) − 0.055 × (upper arm circ.) − 0.023 × (waist circ.) + 0.302 × (ankle circ.) + 0.216 × (outside leg length) − 0.0014 × (surface area leg)	Current study (Eq. #4)
	− 19.279 + 0.474 × age + 0.165 × weight + 0.519 × (ankle circ.) + 0.108 × (outside leg length)	Current study (Eq. #5)

Units of measurement: cm for all circumferences and lengths; cm^2^ for surface areas; cm^3^ for volumes; kg for weight; years for age.

*Circ.* Circumference, *NHOPI* Native Hawaiian and other Pacific Islander.

The following avatar-derived anthropometric and body composition parameters were considered for reproducibility and validity assessments (see below): body surface area, waist circumference, hip circumference, averages of the two sides for the arm, thigh, and calf circumferences, fat mass and its percentage, total lean mass, and appendicular lean mass.

### Statistical analysis

Prior to statistical analyses, outliers of DXA-derived and Mobile Fit app-derived measurements were removed using the Grubbs’ outlier test (alpha = 0.05)^28^ as a part of preprocessing quality control: 2 male players were identified as outliers (1 for DXA-derived measurements, 1 for app-derived measurements) and their measurements removed.

Normality of the data distributions was assessed with the Shapiro–Wilk test and parametric statistical tests (paired sample T test, Pearson correlation analysis) were used.

#### Reproducibility assessment

Changes in anthropometric parameters and body composition measurements between the two avatars were analyzed with the paired sample T test to assess the presence of systematic bias. Measurement reproducibility (i.e., the extent to which scores on repeated measurements—avatar 1 vs avatar 2—are close to each other) was evaluated using: (i) root mean square error (RMSE); (ii) root mean square coefficient of variation (RMS-%CV); (iii) standard error of measurement (SEM) that was calculated as follows: √mean square error term from the Analysis of Variance (ANOVA)/median value between the two avatars): a value < 10% was considered as low (i.e., high agreement between different measurements)^29^; (iv) smallest detectable change (SDC: the smallest individual change in a score that can be interpreted as a real change) that was calculated as follows: 1.96 × √2 × SEM^29^.

#### Validity assessment

The following comparisons between the DXA-derived and Mobile Fit app-derived measurements (the average of the two avatars was considered) were performed: paired sample T test, absolute average differences obtained from the Bland–Altman plots, Pearson correlation analysis, Passing-Bablok regression analysis. The latter analysis was specifically designed to compare two data sets, given by two different methods, both affected by experimental errors^30,31^. If zero is not part of the 95% confidence interval of the intercept of the regression line, it may be concluded that systematic differences exist between the data acquired by the two methods. If 1 is not part of the 95% confidence interval of the slope of the regression line, it may be concluded that proportional differences exist between the compared data sets. For these conclusions to be valid, Passing and Bablok recommended a sample size of at least 50 subjects^30,31^. The Passing-Bablok regression can only be applied if the data are well-fitted by a linear model: therefore, the Cusum test for linearity was also performed.

#### Equation reparameterization and predictors reduction

The fit linear regression model (“*fitlm*” command in Matlab) was used to develop population-specific equations for the body fat percentage estimation (we adopted the same predictors previously identified by Harthy et al.^27^) and the appendicular lean mass estimation (we adopted the same predictors recently identified by McCarthy et al.^25^). Moreover, the stepwise linear regression was also adopted to identify the minimum number of statistically significant appendicular lean mass predictors (among those selected by McCarthy et al.^25^) in order to avoid model overfitting^32,33^.

Data were expressed as median and 1st–3rd quartile and were represented with violin plots showing the probability density functions of the data sets. The threshold for statistical significance was set to P = 0.05. Statistical tests were performed with Matlab (MathWorks, Inc., Natick, MA, USA), MedCalc v. 20.218 (MedCalc Software Ltd, Ostend, Belgium), SPSS v. 20.0 (SPSS Inc., Chicago, IL, USA) software packages.

### Ethical approval

The study conformed to the guidelines of the Declaration of Helsinki and was approved by the ethics committee of the University of Turin (protocol n. 0574321).

## Results

The reproducibility analysis was performed in a total sample of 186 players (image processing errors implied the rejection of the first acquisition for one female player and of the second acquisition for three male players and one female player).

**Figure 2:**
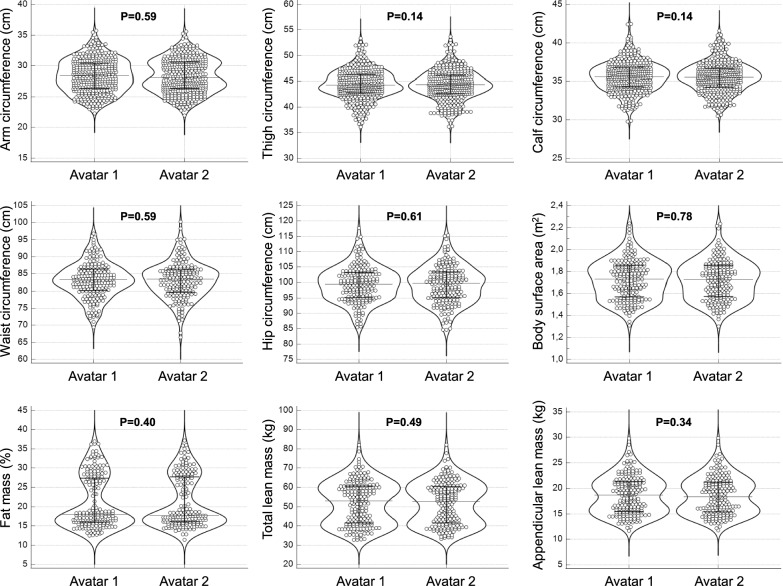
Violin plots of the anthropometric and body composition measurements obtained for the two avatars in the whole group of male and female soccer players. Error bars indicate the median values and the interquartile ranges.

No significant differences were obtained between the two avatars for all anthropometric and body composition measurements (paired T test: P > 0.05 for all comparisons—Fig. 2).

As shown in Table 2, the RMSE values were in the range 0.7–3.4 cm for the circumference measurements (the value obtained for the body surface area was 0.03 m^2^) and in the range 0.3–1.0 kg for the body composition estimates (the value obtained for the body fat percentage was 1.7%). The RMS-%CV values were in the range 1.2–2.6% for the anthropometric measurements and in the range1.1–5.2% for the body composition estimates. The SEM values were < 10% for all anthropometric and body composition measurements. The SDC values were in the range 1.2–6.6 cm for the circumference measurements (the value obtained for the body surface area was 0.06 m^2^) and in the range 0.6–1.9 kg for the body composition estimates (the value obtained for the body fat percentage was 3.3%).

**Table 2:** Reproducibility analysis results.

Variables	Median values (1st–3rd quartile)	RMSE	RMS-CV (%)	SEM (%)	SDC
Arm circumference (cm)	28.3 (26.4–30.5)	0.7	1.8	1.8	1.4
Thigh circumference (cm)	44.3 (42.7–46.3)	1.3	2.0	2.0	2.5
Calf circumference (cm)	35.6 (34.3–36.7)	0.6	1.2	1.2	1.2
Waist circumference (cm)	83.3 (80.0–86.2)	3.1	2.6	2.6	6.0
Hip circumference (cm)	99.5 (95.1–103.4)	3.4	2.3	2.4	6.6
Body surface area (m^2^)	1.73 (1.58–1.85)	0.03	1.08	1.16	0.06
Fat mass (%)	17.8 (16.1–27.5)	1.7	5.2	6.7	3.3
Fat mass (kg)	12.9 (10.9–15.6)	1.0	5.2	5.4	1.9
Total lean mass (kg)	52.9 (41.6–60.5)	1.0	1.6	1.3	1.9
Appendicular lean mass (kg)	18.4 (15.5–21.3)	0.3	1.1	1.2	0.6

*RMSE* root mean square error, *RMS-CV* root mean square coefficient of variation, *SEM* standard error of measurement, *SDC* smallest detectable change.

The agreement between DXA and Mobile Fit app was analyzed in a total sample of 122 male players and 69 female players (after exclusion of 2 outliers).

**Figure 3:**
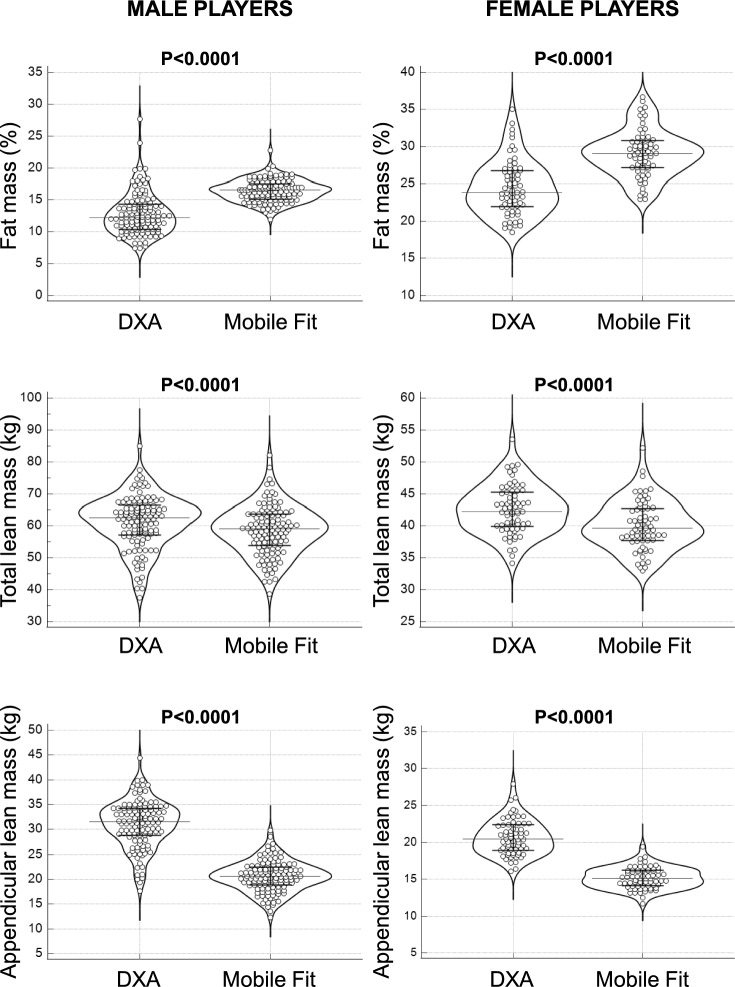
Violin plots of the DXA-derived and Mobile Fit app-derived body composition measurements (data for the two avatars were averaged) obtained in the whole group of male and female soccer players. Error bars indicate the median values and the interquartile ranges.

**Figure 4:**
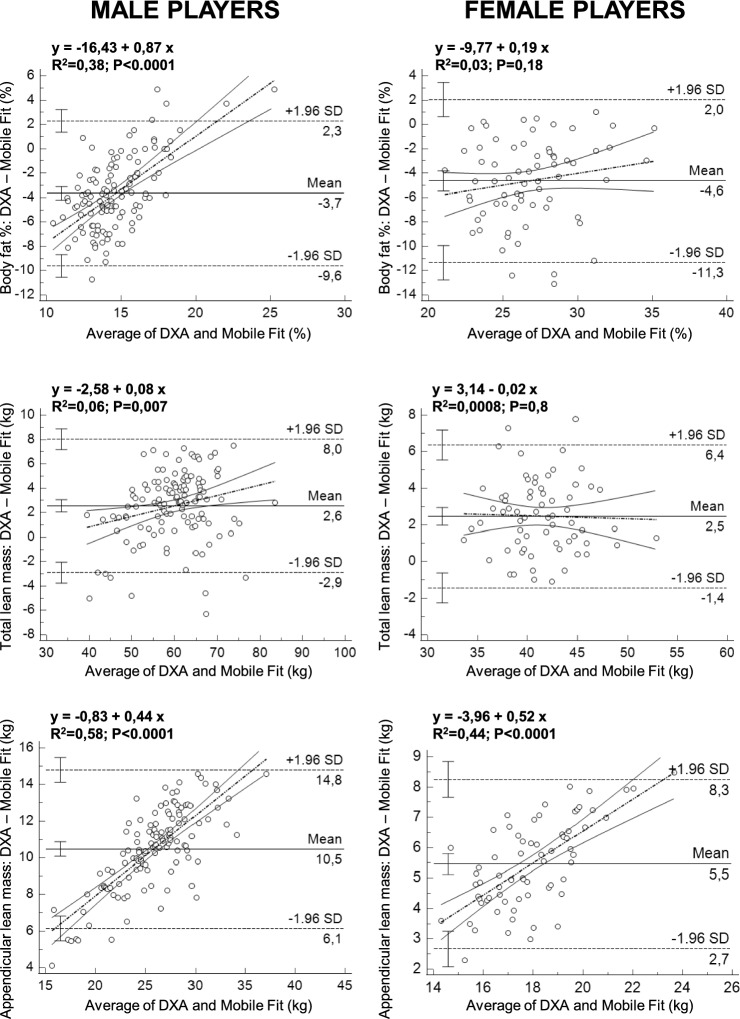
Bland–Altman plots of differences vs. means of the body fat percentage, total lean mass, and appendicular lean mass in male players (left column) and in female players (right column). In each plot, the solid horizontal line depicts the mean of the differences, whereas dashed horizontal lines represent the upper and lower limit of agreement (SD: standard deviation of the differences). The error bar displayed on each horizontal line represents the 95% confidence interval of the corresponding quantity. The dashed-dotted linear regression line (sandwiched between its 95% confidence interval curves) is indicative of proportional bias (whenever its slope is different from zero).

As shown in Fig. 3, the paired T test showed significant differences (P < 0.0001 for all comparisons) between DXA and Mobile Fit app (data obtained for the two avatars were averaged) for all body composition measurements. The Bland–Altman plots (Fig. 4) showed that Mobile Fit app overestimated the body fat percentage with respect to DXA (the average overestimation was + 3.7% in males and + 4.6% in females), while it underestimated the total lean mass (− 2.6 kg in males and − 2.5 kg in females) and the appendicular lean mass (− 10.5 kg in males and − 5.5 kg in females) with respect to DXA.

**Figure 5:**
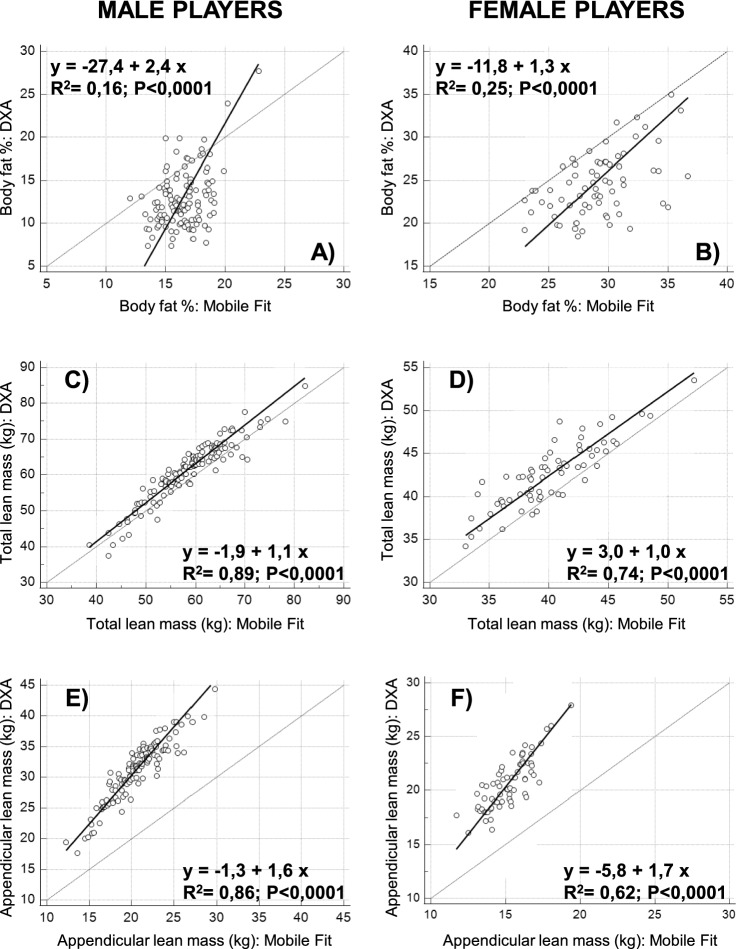
Relations between DXA-derived and Mobile Fit app-derived measurements of body fat percentage, total lean mass, and appendicular lean mass investigated through the Passing-Bablok regression analysis in male players (left column) and in female players (right column). The regression plots include the line of identity and the generated regression line along with the R^2^ and P value (obtained through the Pearson correlation).

As shown in Fig. 5, we found significant positive correlations between DXA and Mobile Fit app for all body composition measurements: the R^2^ values ranged between 0.16 and 0.89 in male players and between 0.25 and 0.74 in female players. As shown in Table 3, the P values obtained by the Cusum test for linearity (range of P values between 0.18 and 0.65) indicated that the Passing-Bablok regression was applicable. The regression analyses in male players showed no systematic differences between DXA and Mobile Fit app for all body composition measurements (Table 3: the 95% confidence intervals of the regression intercepts included the 0 value for all measurements). However, proportional differences between DXA and Mobile Fit app were observed for the body fat percentage and appendicular lean mass (Fig. 5A–E and Table 3: the 95% confidence intervals of the regression slopes did not include the 1 value). The regression analyses in female players showed systematic differences between DXA and Mobile Fit app for the body fat percentage and the appendicular lean mass (Fig. 5B–F and Table 3: the 95% confidence intervals of the regression intercepts did not include the 0 value). Moreover, proportional differences between DXA and Mobile Fit app were also observed for the appendicular lean mass (Fig. 5F and Table 3: the 95% confidence interval of the regression slope did not include the 1 value). The Bland–Altman plots (Fig. 4) confirmed the presence of proportional biases between DXA and Mobile Fit app in both male and female players: in fact, significant (P < 0.0001) positive correlations were obtained between the differences and means for the body fat percentage in male players (i.e., the higher the body fat percentage, the lower the Mobile Fit app overestimation with respect to DXA) and for the appendicular lean mass in both groups of players (i.e., the higher the appendicular lean mass, the higher the Mobile Fit app underestimation with respect to DXA).

**Table 3:** Results of the Passing-Bablok regression analyses.

	Male players (N = 122)	Female players (N = 69)
Body fat %		
Linear model validity		
Cusum test for linearity	P = 0.18	P = 0.29
Systematic differences		
Intercept	27.4	− 11.8
95% confidence interval	42.2 to − 18.0	− 25.8 to − 2.4
Proportional differences		
Slope	2.4	1.3
95% confidence interval	1.9 to 3.3	0.9 to 1.7
Total lean mass (kg)		
Linear model validity		
Cusum test for linearity	P = 0.37	P = 0.65
Systematic differences		
Intercept	− 1.9	3.0
95% confidence interval	− 6.1 to 1.9	− 2.7 to 8.1
Proportional differences		
Slope	1.1	1.0
95% confidence interval	1.0 to 1.2	0.9 to 1.1
Appendicular lean mass (kg)		
Linear model validity		
Cusum test for linearity	P = 0.37	P = 0.29
Systematic differences		
Intercept	− 1.3	− 5.8
95% confidence interval	− 3.8 to 1.0	− 10.3 to − 1.7
Proportional differences		
Slope	1.6	1.7
95% confidence interval	1.5 to 1.7	1.5 to 2.0

**Figure 6:**
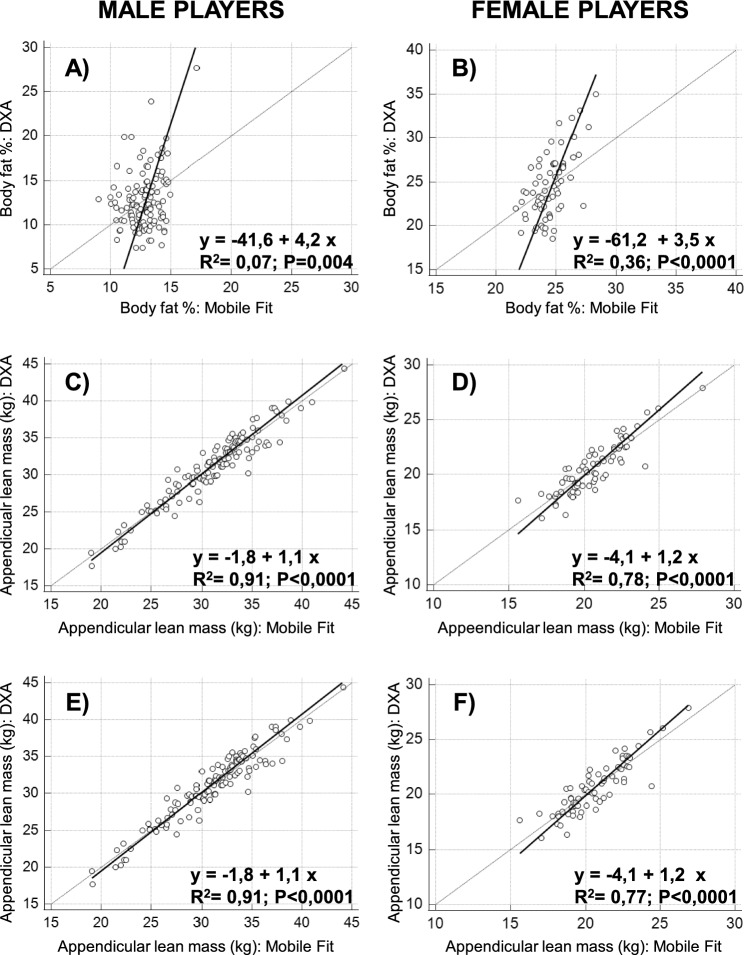
Relations between DXA-derived and Mobile Fit app-derived (after equation reparameterization in top and middle panels and after equation reparameterization and predictors reduction in bottom panels) measurements of the body fat percentage and appendicular lean mass investigated through the Passing-Bablok regression analysis in male players (left column) and female players (right column). The regression plots include the line of identity and the generated regression line along with the R^2^ and P value (obtained through the Pearson correlation).

Using data of the soccer players, we reparameterized the equation previously proposed by Harthy et al.^27^ and we obtained the population-specific equation reported in Table 1 (equation #1). The reparameterization minimized the differences between the DXA-derived and Mobile Fit app-derived estimations of body fat percentage (male players: average difference of 0.0% with min–max differences of − 6.5% and 10.6%; female players: average difference of 0.0% with min–max differences of − 6.2% and 6.7%). However, the Passing-Bablok regression analysis (Cusum tests for linearity: P value of 0.18 in male players and 0.29 in female players) showed systematic and proportional differences in both male players (Fig. 6A: 95% confidence interval of the regression intercept: − 66.2 to − 25.8; 95% confidence interval of the regression slope: 3.0 to 6.1) and female players (Fig. 6B: 95% CI of the regression intercept: − 90.0 to − 42.4; 95% confidence interval of the regression slope: 2.7 to 4.7).

Using data of the soccer players, we reparameterized the equations recently proposed by McCarthy et al.^25^ and we obtained the population-specific equations reported in Table 3 (equation #2 for males and equation #4 for females). The reparameterization minimized the differences between the DXA-derived and Mobile Fit app-derived estimations of the appendicular lean mass (male players: average difference of 0.0 kg with min–max differences of − 4.4 kg and 3.1 kg; female players: average difference of 0.0 kg with min–max differences of − 3.3 kg and 2.1 kg). As shown in Fig. 6C,D, significant positive correlations were obtained between DXA-derived and Mobile Fit app-derived estimations: the R^2^ value was 0.91 for male players (higher than R^2^ of 0.86 of Fig. 5E) and 0.78 for female players (higher than R^2^ of 0.62 of Fig. 5F). The Passing-Bablok regression analysis (Cusum tests for linearity: P value of 0.66 in male players and 0.45 in female players) showed no systematic and no proportional differences in male players (Fig. 6C: 95% confidence interval of the regression intercept: − 3.7 to 0.1; 95% confidence interval of the regression slope: 1.0 to 1.1), while systematic and proportional differences were still observed in female players (Fig. 6D: 95% confidence interval of the regression intercept: − 7.4 to − 1.0; 95% confidence interval of the regression slope: 1.1 to 1.3).

Stepwise linear regression enabled to identify a limited number of appendicular lean mass predictors (shown in Table 1: equation #3 with 6 predictors for males and equation #5 with 4 predictors for females) among those selected by McCarthy et al. (13 predictors for males and 12 predictors for females)^25^. The differences between the DXA-derived and Mobile Fit app-derived estimations of the appendicular lean mass were minimized (male players: average difference of 0.0 kg with min–max differences of − 4.4 kg and 3.1 kg; female players: average difference of 0.0 kg with min–max differences of − 3.7 kg and 2.0 kg). As shown in Fig. 6E,F significant positive correlations were obtained between DXA-derived and Mobile Fit app-derived estimations: the R^2^ values were 0.91 for male players (equal to R^2^ value of panel C) and 0.77 for female players (comparable to R^2^ value of panel D). The Passing-Bablok regression analysis (Cusum tests for linearity: P value of 0.66 in male players and 0.65 in female players) showed no systematic and no proportional differences in male players (Fig. 6E: 95% confidence interval of the regression intercept: − 3.7 to 0.1; 95% confidence interval of the regression slope: 1.0 to 1.1), while systematic and proportional differences were still observed in female players (Fig. 6F: 95% confidence interval of the regression intercept: − 6.9 to − 1.2; 95% confidence interval of the regression slope: 1.1 to 1.3).

## Discussion

In the present study we investigated the reproducibly and validity of smartphone-based estimation of clinically relevant anthropometric and body composition parameters in a large group of youth soccer players. The main results of this study can be summarized as follows: (i) Mobile Fit app provided precise measurements of body size and composition; (ii) Mobile Fit app overestimated the body fat percentage with respect to DXA, while it underestimated the total and the appendicular lean masses in both male and female players; (iii) reparameterization of the equations previously proposed to estimate the body fat percentage^27^ and the appendicular lean mass^25^ minimized the differences between the DXA-derived and Mobile Fit app-derived estimations.

The demonstration of high agreement between the measurements obtained for consecutive avatars confirms previous studies on the clinical application of smartphone-based digital imaging analysis that were performed in healthy adults through different commercially-available tools^19,21–23^. To our knowledge, this study is the first investigating youth soccer players and it is also the first performing smartphone-based analysis in a clinical setting, outside well-controlled laboratory settings. The smartphone app evaluated in this study is similar to the MeThreeSixthy app evaluated in previous studies^19,20,24^: both apps capture a series of bidimensional photographic silhouettes that are then extracted and linked to a tridimensional template mesh using artificial intelligence and machine learning algorithms. Although these apps are easy to use and consecutive scans can easily be acquired, a practical implication of the observed findings is that duplicate (or triplicate) acquisition of front and side images is not mandatory in a clinical setting: if the measurement conditions (i.e., lighting) and the participant attire and pose are appropriate, a single acquisition can provide robust body size measurements. Another implication of the observed high reproducibility of measurements is that the app we evaluated can be used to monitor the body size changes in response to interventions (e.g., training, diet) and the SDC values reported in Table 2 will help clinicians and trainers interpret the clinical meaning of the body circumference and composition changes over time at individual level.

The smartphone app evaluated in this study uses the prediction equation for body fat percentage previously developed by Harthy et al.^27^ through a gold standard four-compartment body composition model. Previous studies demonstrated its accuracy in healthy adults and its underestimation of the body fat percentage in individuals with higher degrees of adiposity (i.e., percentages above 30%)^24,27^. Conversely, we found that the app overestimated the body fat percentage with respect to DXA in both male and female players.

The smartphone app evaluated in this study uses the prediction equation for appendicular lean mass recently proposed by McCarthy et al.^25^ who demonstrated its accuracy with respect to DXA in small groups of adult men and women. Conversely, we found that the app underestimated the appendicular lean mass in both male and female players.

Differences in age and variability in body size and composition between the previously investigated populations and our group of athletes are possible explanations for the discrepancies between the previous^24,25,27^ and the present results. A methodological implication of these findings is that the anthropometrics-based prediction models obtained in healthy adults or in persons with obesity cannot be applied to assess the body composition in young athletes. Consistently, other body composition assessment approaches (e.g., bioimpedance analysis) require the selection of “normal” or “athletic” settings as different prediction models were developed and validated for “normal” and “athletic” subjects.

The reparameterization of the two equations and the predictors reduction for the McCarthy’s equation^25^ were performed as a preliminary effort to develop population-specific equations for the body fat percentage and the appendicular lean mass estimations. Although the reparameterization minimized the differences between the DXA-derived and Mobile Fit app-derived estimations, systematic and proportional differences between the DXA-derived and the app-derived estimations were still observed in male and female players for the body fat percentage and in female players for the appendicular lean mass. Further studies in large groups of soccer players are required to validate in independent data sets the population-specific models presented in this manuscript.

Further studies are also required to develop new models for body composition prediction in young athletes. It is well-known they undergo not only body size but also body shape changes that are underlain by both skeletal growth and sport-specific muscle size adaptations. For instance, soccer players tend to have an hourglass body shape with a larger proportion of their appendicular lean mass in the proximal region of the lower extremities (i.e., anterior and posterior thigh). It can therefore by hypothesized that body shape-based models could be required in young athletes to capture information about body composition beyond conventional anthropometric measurements, as it has already been observed in healthy adults^34–36^.

This study has several limitations. First, only one smartphone app was adopted for digital anthropometric assessment of soccer players: therefore, our results (high reproducibility of the app-derived measurements, app-derived overestimation of the body fat percentage and underestimation of the appendicular lean mass with respect to DXA) and the anthropometrics-based prediction models presented in Table 1 are device-specific and are not generalizable beyond the MeThreeSixthy and Mobile Fit apps. Second, we assessed the agreement between the measurements obtained for consecutive avatars, but the short-term and long-term reproducibility of the measurements was not investigated. Third, we used DXA instead of gold-standard approaches (i.e., four-compartment model for the body fat percentage estimation and whole-body magnetic resonance imaging for appendicular lean mass estimation) to derive body composition estimations used to perform the validity assessments. Fourth, we did not control for factors that might influence body composition (such as menstrual cycle phase in female players, hydration status in both male and female players) and might thus add variability to measurements and predictions.

In conclusion, this study showed that the digital anthropometric assessment with a smartphone app in youth soccer players provided precise measurements of body size and composition. Previously proposed anthropometrics-based prediction models obtained in adults cannot be applied to assess body composition in young athletes: population-specific models are therefore required given their body composition and shape features.

## Data Availability

Data described in this manuscript will be made available upon request and approval by the principal investigator, Marco A. Minetto (marco.minetto@unito.it).

## Abbreviations

ANOVA: Analysis of variance
DXA: Dual-energy X-ray absorptiometry
RMS-%CV: Root mean square coefficient of variation
RMSE: Root mean square error
SDC: Smallest detectable change
SEM: Standard error of measurement
